# “Let Me Tell You About My…” Provider Self-Disclosure in the Emergency Department Builds Patient Rapport

**DOI:** 10.5811/westjem.2016.10.31014

**Published:** 2016-11-23

**Authors:** Korie L Zink, Marcia Perry, Kory London, Olivia Floto, Benjamin Bassin, John Burkhardt, Sally A Santen

**Affiliations:** * University of Michigan, University of Michigan Medical School, Ann Arbor, Michigan; † University of Michigan, Department of Emergency Medicine, Ann Arbor, Michigan; ‡ University of Michigan, Ann Arbor, Michigan; § University of Michigan, Department of Learning Health Sciences, Ann Arbor, Michigan

## Abstract

**Introduction:**

As patients become increasingly involved in their medical care, physician-patient communication gains importance. A previous study showed that physician self-disclosure (SD) of personal information by primary care providers decreased patient rating of the provider communication skills.

**Objective:**

The objective of this study was to explore the incidence and impact of emergency department (ED) provider self-disclosure on patients’ rating of provider communication skills.

**Methods:**

A survey was administered to 520 adult patients or parents of pediatric patients in a large tertiary care ED during the summer of 2014. The instrument asked patients whether the provider self-disclosed and subsequently asked patients to rate providers’ communication skills. We compared patients’ ratings of communication measurements between encounters where self-disclosure occurred to those where it did not.

**Results:**

Patients reported provider SD in 18.9% of interactions. Provider SD was associated with more positive patient perception of provider communication skills (p<0.05), more positive ratings of provider rapport (p<0.05) and higher satisfaction with provider communication (p<0.05). Patients who noted SD scored their providers’ communication skills as “excellent” (63.4%) compared to patients without self-disclosure (47.1%). Patients reported that they would like to hear about their providers’ experiences with a similar chief complaint (64.4% of patients), their providers’ education (49%), family (33%), personal life (21%) or an injury/ailment unlike their own (18%). Patients responded that providers self-disclose to make patients comfortable/at ease and to build rapport.

**Conclusion:**

Provider self-disclosure in the ED is common and is associated with higher ratings of provider communication, rapport, and patient satisfaction.

## INTRODUCTION

Strong communication skills are crucial to effective interactions with patients in the emergency department (ED).^1^ As patient-centered care and shared decision-making become central to medicine, effective physician communication continues to gain importance. Successful communication increases patient and physician satisfaction.^2^ Several studies have demonstrated that high satisfaction levels correlate with medical compliance, return to the same ED for future care, and increased referral of others to that ED.^3^–^6^ Given the time constraints of ED interactions, some providers use self-disclosure (SD), or the sharing of personal information and/or details of their experiences, to gain trust and build rapport with their patients. One article suggested that patients do not respond favorably to doctors who show vulnerability, which is a risk during physician SD.^7^ Other studies maintain that establishing robust physician-patient relationships are health-promoting and that SD may play a role.^8^ A recent perspective in *New England Journal* discussed the tension between developing rapport and the observation that “sharing personal experiences exposes both our biases and our vulnerabilities, which may not be socially, professionally, or emotionally safe.”^9^

Self-disclosure is somewhat controversial, as some patients may appreciate personal anecdotes while others find them irrelevant or intrusive to their care. Previous studies have found mixed results regarding the influence of SD on patient satisfaction in primary care and surgical settings.^10^–^12^ This suggests that clinical setting may have an impact on patient perception of SD. The effects of physician SD on patient-doctor relationships in the ED have not yet been studied.

The objective of this study was to determine the incidence of provider SD and explore the impact of ED provider SD on patients’ assessment of physician communication skills. Specifically, we investigate whether provider SD increases or decreases patients’ assessment of providers’ communication and rapport.

## METHODS

### Study Design, Population, and Setting

We conducted this observational, cross-sectional, mixed-methods survey in the adult and pediatric EDs of an academic Level 1 tertiary hospital. The institutional review board determined this study to be exempt. Surveys were administered between April and July of 2014 by trained student research assistants. The study population consisted of a convenience sample of adult patients or parents of pediatric patients. We excluded patients if they could not communicate effectively in English, were critically ill, or cognitively impaired.

### Study Protocol

Patients were approached for the study after they had been evaluated by a medical provider (an attending, a resident, or a physician assistant). Patients were enrolled after verbal consent and informed that their individual results would not be shared with providers or in any way affect their care. Patients were shown a picture of their care provider and completed the written survey for this provider. If patients were unable to complete the survey, either the research assistant or family member assisted in completion. Patients were not aware of the purpose of the study before their encounters with providers. Providers were not made aware of the purpose of the study until the data collection was complete.

### Measurements

The survey is provided in the Appendix A. For validity purposes, researchers performed a literature review, and the survey was designed to cover topics frequently mentioned in previous studies.^9^–^12^ Further, we modeled the questions after those used by Beach.^12^ Demographic data were collected. Patients also indicated whether or not their providers self-disclosed and, if so, the content of SD. Specifically, to indicate SD patients were asked “Did your doctor talk about herself/himself today?” The instrument used a Likert-type five-point scale to rate communication skills, rapport building, and satisfaction with communication (Table 1, Appendix A). The remaining questions asked patients whether or not they would like to have their ED or primary care provider (PCP) talk about her/his educational background, family, social life, medical ailments or injuries. Finally, patients were asked how likely they were to follow their providers’ medical recommendations. The survey was piloted to 20 patients to collect validity evidence (response process and internal consistency) and discussed with patients to ascertain points of confusion. Two questions were subsequently revised.

The outcomes were frequency of reported physician SD and patients’ ratings of provider communication, rapport, and satisfaction with communication skills. We compared patients’ ratings of these communication measurements between encounters where the provider self-disclosed and encounters where the provider did not.

### Data Analysis

We performed descriptive statistics. The Likert-type ordinal data were analyzed using nonparametric Kruskal-Wallis tests to investigate the relationship between the providers’ SD and patient ratings of provider communication, rapport, and satisfaction with communication (SPSS 19). We estimated a multinomial logistic regression with outcomes of below average to average (1–3), good (4), and very good (5) communications scores. Independent variables were provider role and whether the provider talked about her/himself (STATA 12). Answers to open-ended questions were coded for frequencies of response using qualitative analysis to develop categories.^13^ We used qualitative thematic analysis approach, in which a single author read iteratively through the comments. Codes were generated inductively according to a reading and rereading of the primary data. Once the primary codes were determined, all of the comments were coded accordingly.

## RESULTS

During the study period, 520 patients completed the survey. The mean age was 44 years old; 55% were female, and 59% had an education level greater than a high school diploma/GED. Of the 520 patients surveyed, 18.9% indicated that their provider talked about her/himself during their ED visit, 69.8% said that there was not SD, and 11.3% were unsure whether or not their providers self-disclosed. When we examined SD of each provider, nearly half of 84 physicians (52.4%) self-disclosed information during at least one encounter. Further, patients felt it was important to build a good relationship with their ED care providers, with 96% responding “very important” or “somewhat important.” Table 1 shows patient ratings of provider communication skills, rapport, and satisfaction with communication, which are the outcome variables.

Encounters with SD were rated more highly than encounters where the provider did not self-disclose. Provider SD was associated with more positive patient ratings of provider communication skills (p<0.05), more positive ratings of provider rapport (p<0.05), and higher satisfaction with provider communication (p<0.05) (Table 1). Patients who noted provider SD scored their providers’ communication skills as excellent 63.4% of the time compared to patients without SD 47.1% of the time. Patients who noted provider SD were “very satisfied” with the providers’ communication skills 72.5% of the time, compared to 59.1% without SD. Both pain and reason for presenting to the ED (with a new versus recurrent problem) were not statistically significant variables in single variable regression analysis with any of our outcomes (rapport, communication score, or satisfaction with provider communication skills).

Patients were asked what the physicians disclosed. SDs followed several themes, including casual conversation (28.6%), rapport building (23.4%), reassurance (20.8%), humor (14.3%), counseling (6.5%), and extended narratives (6.5%). An example of a casual SD was: “I just bonked my head a few minutes ago.” One physician built rapport by sharing that she had family in the same state that the patient was from. An SD used to reassure a patient was: “I am 34 years old; I’ve been an emergency physician for seven years.” Several physicians used humor, such as: “I like your nail polish. You don’t want to see my toes after I do them, they look horrible!” Some physicians self-disclosed while counseling patients: “I used to have these premature heart beats a lot. I cut back on my caffeine intake...” The extended narratives SDs typically involved stories about the physician’s children. More examples can be found in Appendix B.

Providers who talked about themselves were more likely to score very good (5) on patient perception of communication skills even when considering different provider role (p<0.05). Providers who did not self-disclose had an increased relative risk of 4.2 (95% CI 1.2, 14.2) to score as very poor to adequate (Table 2). This result means that the relative risk ratio of a “very poor to adequate” score relative to a “very good” score is expected to change by a factor of 4.2 when no SD was provided given the other variables in the model are held constant. The reported results are relative to each other and not absolute odds. There was no statistically significant difference between provider roles when included with SD as a factor (Table 2).

When asked how likely respondents were to follow their provider’s medical recommendations, 89.5% of patients indicated very likely, 9.3% somewhat likely, and 1.2% were not likely to follow recommendations. There were no significant differences in intentions to follow medical recommendations between the groups that did and did not experience SD.

Of the patients who experienced SD, 61% said they liked it, 29% did not care, and 7% disliked the SD. For patients who did not experience SD, 27% said that they thought they would like it, 53% said they would not care, and 13% said they would have disliked it if their provider self-disclosed. With regard to specific types of SD, patients indicated a preference of some types of information over others (Figure). Almost two-thirds of patients reported that they would like to hear about their providers’ experiences with a similar ailment/injury to their chief complaint. Patients also were interested in hearing about their providers’ education and family. Patients were less interested in hearing about a provider’s personal life or about an injury/ailment unlike their own. A multivariate test of means demonstrated a significant difference between at least one of these question responses and the others (p<0.05). When asked the benefits of SD, patients responded that providers self-disclose to make patients comfortable/at ease and to build rapport. To gauge whether or not patients would want to know similar information about their ED providers as their PCPs, patients indicated the type of information they would want to know about both types of providers (Table 3). Patients indicated that they preferred to know more about their PCP than their ED provider (p<0.05). Individual patient comments indicated that providers self-disclose to make patients comfortable/at ease and to build rapport (Table 4).

## DISCUSSION

In social contexts, SD is often used to build rapport, make connections, and to try to relate to those around us. In the ED, provider SD is associated with significantly higher patient ratings of provider communication skills, rapport, and satisfaction with communication. Although relationships in the ED are often brief and usually without a previous ongoing relationship, the majority of patients in this sample think that it is very important to build a good relationship with their ED provider. This suggests that even when patients perceive their medical conditions to be acute, they often want to be taken care of by someone they feel they can trust and who will put effort into building rapport with them.

Though extensive research exists regarding communication patterns between physicians and patients, little is known about the influence of provider SD in the ED. Previous work by Beach found that physician SD in a primary care setting is negatively correlated with patient satisfaction, while SD in a surgical setting is positively correlated with patient satisfaction.^11^ The ED can be thought of as an environment encompassing features of both the PCP office and surgical clinic. Many patients present for acute care, while others use the ED as a resource to help manage chronic illness or minor medical problems. Our findings suggest that for patients, the experience of being in the ED may be more akin to those found in surgical clinic settings in terms of the communication expected of medical providers. Within the multivariate analysis, provider role was included and not found to be a significant factor when SD was considered.

Prior research has demonstrated that strong communication skills are associated with effective ED interactions^1^ and that provider empathy has a positive relationship to medical outcomes.^14^ In our study, patients recognized that providers used SD to make the patient comfortable and build rapport. While a few patients thought that a provider might self-disclose because of arrogance or insecurity, the vast majority saw it as evidence of a provider trying to communicate more effectively with the patient by building a relationship.

The high communication and rapport ratings indicate that ED patients are generally happy with their experience. When patients were asked how they felt if their provider talked about him/herself, the majority liked the experience. Patients whose physician did not self-disclose were asked the same question. Those who had conversations with doctors who disclosed information about themselves generally liked it, but those who did not have those conversations did not seem to miss the experience.

Despite the acute or anxiety-provoking circumstances of many ED visits, patients are interested in hearing about providers’ personal experiences. The information that patients want to learn about their ED providers tends to fall into two main categories: (1) provider education and training background, and (2) a provider’s personal experience with a medical ailment/injury that is similar to that of the patient. Many patients felt that hearing a personal story of medical injury from their provider could help them make decisions about their care and demonstrates a more personal touch. Patients are not as interested in hearing about ED providers’ families, their personal/social life, or unrelated personal medical history.

Based on these findings, we would encourage ED providers to think of SD as a potential tool to build rapport, put patients at ease and communicate effectively. However, not all personal topics may be received positively, and effective SD may include provider educational background or similar medical experiences to help build patient confidence and comfort. Dr. Curran recommends that “by asking simple questions— What is my purpose in making this disclosure? How could it benefit my patient? Could it hurt our relationship? —and answering truthfully, we can weigh the risks and benefits within the context of the particular physician–patient relationship.”^9^ Further studies might investigate effective use of SD without over-sharing as well as when providers decide to SD.

## LIMITATIONS

This study has several limitations. First, although patients were told that their providers would not receive results of their surveys, patient responses may have been affected by the fact that they were still cared for by the providers at the time of survey completion and thus may have felt uncomfortable responding. In addition, while we attempted to administer the survey towards the end of the encounter, it is possible that the provider interacted with the patient after the survey was administered and this interaction might have included further SD and affected the patients’ assessment of communication. We did not collect the number of patients who refused to participate. The rate was low, but these refusals may have provided bias. Further, it is unclear whether survey responses were an accurate reflection of providers’ interaction with patients or a composite evaluation of the entire care team or be related to other confounding variables. Attempts to mitigate this included using pictures of providers to specifically prompt patient recall and using questions focused on single providers. Additionally, there may be some recall bias with the patient not remembering exactly what was said. This is further demonstrated in some patients marking that they were not sure if there was SD. Finally, this study was performed at a single site and may not be representative of other EDs.

## CONCLUSION

In summary, providers self-disclose in about 20% of encounters. Self-disclosure in the ED was associated with higher ratings of provider communication, rapport and higher patient satisfaction ratings. Patients are most interested in SDs that relate to their presenting ailment/injury.

## Supplementary Information





## Figures and Tables

**Figure:**
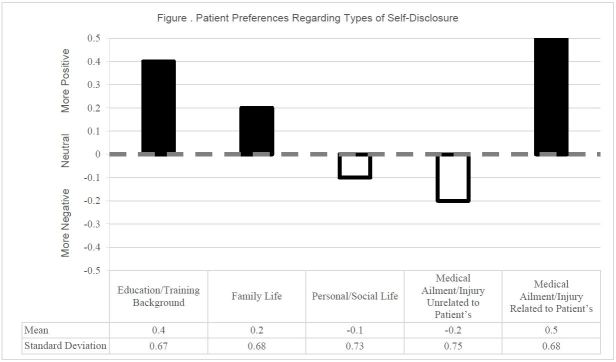
Patient preferences regarding types of self-disclosure. Multivariate tests of means completed by encoding categorical responses (1, 0, −1). P<0.05, meaning there is a statistically significant difference between at least one of the means compared to the others. Mean scores above 0 had more positive responses, mean scores below 0 had more negative responses.

**Table 1:** Patient ratings of provider interaction in the emergency department (SD = Self-Disclosure).

	Presence of SD	Unsure of SD	No SD noted
Provider communication skills	n = 82*	n = 43	n = 314*
Excellent	52 (63.4%)	27 (63.8%)	148 (47.1%)
Very good	27 (32.9%)	15 (34.9%)	129 (41.1%)
Adequate	0 (0.0%)	1 (2.3%)	32 (10.2%)
Poor	2 (2.4%)	0 (0.0%)	3 (1.0%)
Very poor	1 (1.2)	0 (0.0)	2 (0.6)
Provider rapport, No. (%)	n = 82*	n = 43	n = 309*
Excellent	44 (53.7%)	19 (44.2%)	115 (37.2%)
Very good	30 (36.6%)	22 (51.2%)	139 (45.0%)
Adequate	6 (7.3%)	2 (4.7%)	50 (16.2%)
Poor	1 (1.2%)	0 (0.0%)	3 (1.0%)
Very poor	1 (1.2%)	0 (0.0%)	2 (0.6%)
Importance of building good relationship with provider	n = 95	n = 57	n = 349
Very important	68 (71.6%)	36 (63.2%)	244 (69.9%)
Somewhat important	25 (26.3%)	19 (33.3%)	87 (24.9%)
Not at all important	2 (2.1%)	2 (3.5%)	18 (5.2%)
Satisfaction with provider communication skills	n = 91*	n = 54	n = 337*
Very satisfied	66 (72.5%)	39 (72.2%)	199 (59.1%)
Satisfied	21 (23.1%)	14 (25.9%)	108 (32.0%)
Neutral	0 (0.0%)	0 (0.0%)	25 (7.4%)
Dissatisfied	2 (2.2%)	0 (0.0%)	2 (0.6%)
Very dissatisfied	2 (2.2%)	1 (1.9%)	3 (0.9%)

* p<0.05 comparing self-disclosure and no self-disclosure.

**Table 2:** Communication score related to self-disclosure by emergency department provider.

Very Poor to Adequate				Good		
	
Constant: very good, attending, did self disclose	Logit coefficient	Relative risk ratio	95 % confidence interval	Logit coefficient	Relative risk ratio	95 % confidence interval
Physician assistant	−13.66 (547.7)	0.00 (0.00)	−1,087, 1,060	−0.26 (0.48)	0.77 (0.37)	−1.20, 0.68
Resident	−0.75 (0.57)	0.47 (0.27)	−1.86, 0.37	−0.56 (0.33)	0.57 (0.19)	−1.20, 0.09
Did not self-disclose	1.43* (0.63)	4.16* (2.61)	0.19, 2.66	0.60* (0.29)	1.81* (0.51)	0.04, 1.15
constant	2.50	0.08		0.54	0.58	

Standard errors in parentheses

* p<0.05

**Table 3:** Preferred self-disclosure content for emergency department (ED) provider vs primary care provider (PCP).

What information would you want to know about your doctor	ED (%)	PCP (%)
Education/training background	43.9%	58.6%*
Family life	7.6%	26.3%*
Personal/social life	6.1%	18.6%*
Medical ailment/injury unrelated to patient’s	13.1%	21.6%*
Medical ailment/injury related to patient’s	34.5%	39.6%*
Would not like to know anything about provider	36.9%	20.4%

* p<0.05

**Table 4:** Themes of open-ended responses in a study of the effect of provider self-disclosure on patient satisfaction.

How would you/did you feel if your doctor talked about herself/himself regarding other topics not covered during your visit today? Why?^*^	n = 300 Comments
Would/did like it	
Generally positive (makes patient feel better, more personal, humanizing, good communication)	129 (43.0%)
Rapport/relationship/trust building	48 (16.0%)
Makes patient comfortable/more at ease	29 (9.7%)
Patient is interested to know more about provider	13 (4.3%)
Would/did not care	
Depends on nature of SD/don’t know	12 (4.0%)
Would/did dislike it	
Irrelevant/poor use of time	31 (10.3%)
Generally negative	23 (7.7%)
Why do you think a doctor might talk about her/himself?^*^	n = 369
To make patient comfortable/at ease	119 (32.2%)
To build rapport/relationship	80 (21.7%)
To connect/relate/empathize with patient	62 (16.8%)
To educate/share experiences	39 (10.6%)
To build trust/prove credibility	23 (6.2%)
Doctor arrogance/insecurity/just to chat	26 (7.1%)
