# Supplementary material for: “Let Me Tell You About My…” Provider Self-Disclosure in the Emergency Department Builds Patient Rapport
Source: West J Emerg Med. 2016 Nov 23;18(1):43–9. doi: 10.5811/westjem.2016.10.31014 (PMC5226762; doi:10.5811/westjem.2016.10.31014)
Supplement: Supplementary file 1 [file wjem-18-43-s001.docx]

**APPENDIX

Survey of Provider Communication**

| Date: | Time of Survey Completion: | |
| --- | --- | --- |
| Chief Complaint (based on ED chart): | | Bay: |

| Age: | Gender: (Circle) M F Other Decline to Answer |
| --- | --- |
| Race/ ethnicity  (Check all that apply) |  Black or African American White   Asian Native Hawaiian / Other Pacific Islander   Hispanic or Latina/o Decline to Answer   Other |
| Highest  Education Level  Attained |  Less than high school diploma/GED High school diploma/GED   Bachelor’s Degree Graduate Degree   Decline to Answer Other: ___________________ |

| What brings you to the ER today? | | | |
| --- | --- | --- | --- |
| Is this a completely new health problem or a recurrence of a previous health problem? | |  Completely new problem   Recurrence of existing problem | |
| How much pain are you in? Not much pain >>> 0 1 2 3 4 5 6 7 8 9 10 <<< Worst pain you have ever felt? | | | |
| How important is it for you to build a good relationship with your doctor today? | | |  Not at all important   Somewhat important   Very important |
| Please evaluate your doctor’s communication skills.   Excellent   Very Good   Adequate   Poor   Very Poor | Please evaluate the rapport (relationship) you had with the doctor   Excellent   Very Good   Adequate   Poor   Very Poor | | |
| Did your doctor talk about herself/himself today? | | |  Yes   No   I’m not sure |
| If yes, what did your doctor say about him/herself? | | | |
| How would you/did you feel if your doctor talked about herself/ himself regarding other topics not covered during your visit today? | | |  I would/did not like it.   I would/did not care.   I would/did like it. |
| Why? (Optional) | | | |
| How would you /did you feel if your doctor talked about her/his education or training today? | | |  I would/did not like it.   I would/did not care.   I would/did like it. |

| How would you/did you feel if your doctor talked about her/his family today? | |  I would/did not like it.   I would/did not care.   I would/did like it. |
| --- | --- | --- |
| How would you/did you feel if your doctor talked about her/his personal/social life (not related to your medical issues) today? | |  I would/did not like it.   I would/did not care.   I would/did like it. |
| How would you/did you feel if your doctor talked about her/his own medical ailments/injures that was unrelated to your medical issue today? | |  I would/did not like it.   I would/did not care.   I would/did like it. |
| How would you/did you feel if your doctor talked about his  /her medical conditions to help you understand your own  medical condition? | |  I would/did not like it.   I would/did not care.   I would/did like it. |
| Why do you think a doctor might talk about her/himself during a visit with a patient? | | |
| How satisfied were you with your doctor’s communication skills? | |  Very dissatisfied   Dissatisfied   Neutral   Satisfied   Very satisfied |
| What information would you want to know about your doctor in the Emergency Department? (Check all that apply.)   Educational background/ medical training   Family life   Personal/social life   Experiences with ailment/injury  unrelated to my own   Experiences with the same ailment/injury that I have   Other:  ____________________________   I would not like to know anything about my doctor. | What information would you want to know about your Primary Care Physician? (Check all that apply.)   Educational background/ medical training   Family life   Personal/social life   Experiences with ailment/injury unrelated to my own   Experiences with the same ailment/injury that I  have   Other: _____________________________   I would not like to know anything about my doctor. | |
| If your doctor makes medical recommendations for you today (for example, about medications, activities, or follow-up appointments), how likely are you to follow those recommendations? |  Not likely   Somewhat likely   Very likely | |
