# Supplementary material for: “Let Me Tell You About My…” Provider Self-Disclosure in the Emergency Department Builds Patient Rapport
Source: West J Emerg Med. 2016 Nov 23;18(1):43–9. doi: 10.5811/westjem.2016.10.31014 (PMC5226762; doi:10.5811/westjem.2016.10.31014)
Supplement: Supplementary file 2 [file wjem-18-43-s002.docx]

**Appendix B**

**Examples of SDs in Categories**

| **Casual (28.6%)** |
| --- |
| “My daughter loves rhubarb.” |
| “I just bonked my head a few minutes ago.” |
| “I need a financial advisor. I need a shopper, too.” |
|  |
| **Counseling (6.5%)** |
| “I used to have these premature heart beats a lot.  I cut back on my caffeine intake. I used to drink it a lot. Then there were days when I wouldn't have the beats at all.” |
| "I am worried because I saw a person with a stroke the other day and just numbness, tingling." |
| “In my practice, I don't radiate young women unless it's serious.  I don't take something like that lightly.” |
|  |
| **Extended Narrative (6.5%)**  Narrative regarding where the physician went to college and his favorite sports teams.  Narrative about traveling to Boston, including experiences at certain hotels and restaurants.  Narrative about children attending certain schools |
|  |
| **Humor (14.3%)** |
| "If my ankles look this good at age 82, I'll be a happy guy. Heck, if I make it to 82 I'll be a happy guy." |
| "I like your nailpolish. I'm not coordinated, you don't want to see my toes after I do them, they look horrible." |
| "That's the same thing my wife says (that she's only my wife 'sometimes')!" |
|  |
| **Rapport Building (24.4%)** |
| **“**I like your blue toes. My little guy had his painted orange this weekend.” |
| **“**I had that experience with that medication, too.  It's a horrible sensation.”  “"I have family near Minneapolis, too. We used to go there when I was younger. It's a nice area."” |
| **Reassurance (20.8%)** |
| "I've been doing this for 20 years. This is only the third time I've seen this"  "I'm 34 years old, I've been an Emergency Physician for 7 years."  "It's where I get my primary care, to tell you the truth." |
